# Prevalence and causes of visual impairment among older persons living in low-income old age homes in Durban, South Africa

**DOI:** 10.4102/phcfm.v12i1.2159

**Published:** 2020-06-10

**Authors:** Khathutshelo P. Mashige, Serela S. Ramklass

**Affiliations:** 1 Department of Optometry, Faculty of Health Science, University of KwaZulu-Natal, Durban, South Africa; 2 Department of Clinical Medicine, Faculty of Health Science, University of KwaZulu-Natal, Durban, South Africa

**Keywords:** low-income old age home, prevalence, visual acuity, visual impairment, blindness, Durban

## Abstract

**Background:**

Visual impairment (VI) increases with age and has been reported to be more prevalent among older adults living in old age homes than in the general population.

**Aim:**

To determine the prevalence and causes of VI among older adults living in low-income old age homes in Durban, South Africa.

**Setting:**

This study was conducted at low-income old age homes in Durban.

**Methods:**

This cross-sectional study of 118 residents aged 60 years and older, collected socio-demographic data, presenting visual acuities (VAs) for each eye, and binocularly. Anterior segment eye examinations were conducted with a penlight torch and a portable slit-lamp, while posterior segment evaluation was conducted with direct and indirect ophthalmoscopy. Objective and subjective refractions were performed, and the best-corrected distance and near VAs were measured in each eye. VI was defined as presenting VA < 6/18 and included moderate VI (< 6/18−6/60), severe VI (< 6/60 –3/60) and blindness (< 6/120).

**Results:**

The mean age of the participants was 73.3 years and included 80.5% females and 19.5% males. The prevalence of VI and blindness was 63.6%. Optical correction significantly reduced the prevalence of VI and blindness by 19.5% (*p* < 0.05). The main causes of non-refractive VI and blindness were cataract (54.5%), posterior segment disorders (25.5%) and corneal opacities (20%).

**Conclusion:**

The prevalence of VI and blindness is high among residents in low-income old age homes living in Durban. Refractive correction and surgical cataract intervention can significantly reduce the burden of VI and blindness among the elderly residents.

## Introduction

The World Health Organization (WHO) defines visual impairment (VI) as presenting visual acuity (VA) worse than 6/18, but better and equal to 3/60 or a corresponding visual field (VF) loss of less than 20 degrees around the central fixation in the better eye, with presenting optical correction, if any.^1^ Persons with presenting VA of < 6/18−6/60 in the better-seeing eye are said to have moderate VI and those with presenting VA of < 6/60–3/60 have severe VI.^1^ Blindness is defined as presenting VA of worse than 3/60, or a corresponding VF loss of less than 10 degrees around the central fixation in the better eye, with presenting optical correction, if any.^1^ Globally, there are an estimated 1.3 billion people who live with VI, majority of whom are over the age of 50 years.^2^ The principal causes of VI among the global population in 2015 were uncorrected refractive errors (53.69%), cataract (24.28%), age-related macular degeneration (3.88%), glaucoma (1.85%) and diabetic retinopathy (1.20%), while the leading causes of blindness were cataract (35%), uncorrected refractive error (20.56%) and glaucoma (8.06%).^2^ Among adults aged 50 years and older in 2015, the contribution of each cause to VI was the following: uncorrected refractive errors (52.34%), cataract (25.15%), glaucoma (2.05%), age-related macular degeneration (4.38%), diabetic retinopathy (1.30%), corneal opacity (1.14%), trachoma (0.64%) and other (13%), while uncorrected refractive errors, cataract, glaucoma, age-related macular degeneration, diabetic retinopathy, corneal opacity, trachoma and other contributed 20.28%, 35.15%, 8.49%, 5.93%, 1.06%, 3.21%, 0.97% and 24.92% of blindness, respectively.^2^ Globally, cataract and uncorrected refractive error combined contributed 77% of VI and 55% of blindness in adults aged 50 years and older in 2015.^2^

In South Africa, the prevalence of moderate and severe VI was reported to be 2.0% (662 472 of 50.1 million) in 2010.^3^ Cockburn et al.^4^ reported that in 2012, the prevalence of VI and blindness among people ≥ 50 years in Cape Town was 4.9% and 1.4%, respectively. Refractive errors were the leading cause of VI (50%) and severe VI (22%), while cataracts were the leading single cause of severe VI (37%) and the second leading cause of blindness (27%).^4^ Diabetic retinopathy was responsible for 2%, 11% and 8% of VI, severe VI and blindness, respectively.^4^ Glaucoma accounted for 6%, 7% and 11% of VI, severe VI and blindness, respectively.^4^ Avoidable causes, which included cataract, refractive error, trachoma and other causes of corneal scars, made up 79%, 63% and 35% of VI, severe VI and all blindness, respectively.^4^

The prevalence of VI and blindness increases with age because of the high frequency of irreversible (such as glaucoma, age-related macular degeneration and other retinal diseases) and reversible diseases (such as cataract) among older adults.^5,6,7^ Several studies^8,9,10^ have reported a high prevalence of VI and blindness among older adults in old age homes in the United States and other industrialised countries. The prevalence rates have been reported to be 3–15 times higher than the same age of the base population living outside old age homes.^11,12^ The above authors^11,12^ suggested that these differences are a result of those residing in old age homes often not having the same access to healthcare as persons living at home, and that there is a shortage of eye care professionals who routinely serve people living in old age homes.

The health, social and economic implications of VI and blindness for both the sufferers and their country have been described in many publications.^13,14,15,16,17,18,19^ For instance, VI is associated with poor quality of life (QOL), lack of independence and mobility, and has been linked to falls, injury and worsened status in mental health, cognition, social function, employment and educational attainment.^20,21^ The WHO and the International Agency for the Prevention of Blindness (IAPB), with an international membership of non-governmental organisations (NGOs), professional associations, eye care institutions and corporations, developed the global Vision 2020 initiative, the Right to Sight Campaign, to eliminate preventable blindness by 2020. This initiative has to date successfully reduced world blindness by approximately 15 million.^22^ However, the WHO’s report on ‘Universal Eye Health: a global action plan 2014–2019’ indicated that many people with VI globally were still undetected or untreated. It is therefore important to conduct regional studies across the world to understand the epidemiology of VI and blindness and its associated burdens, and to plan strategies to address them.

Although it is well documented^4,23,24^ that the frequency of VI and blindness increases among older persons, there is no documented work about the magnitude of this problem among this group in South Africa, some of whom reside in old age homes. An understanding of the vision status and ocular disease patterns among older adults living in old age homes provides an opportunity for implementing appropriate interventions that can significantly reduce VI and its associated burden in this group. As part of a comprehensive assessment of the vision status and ocular health of older adults in a multiracial community, we determined the prevalence and causes of VI and blindness in this group residing in old age homes in Durban, South Africa. To the best of our knowledge, this is the first survey in the region to examine the prevalence of VI and blindness in this cohort.

## Research methods and design

### Study design

This was a descriptive, cross-sectional, community-based study conducted among the older adults living in five low-income old age homes of Durban.

### Setting

The five low-income old age facilities included in the study were The Association for the Aged (TAFTA) on Ridge, TAFTA Ray Hulette, TAFTA Mary Asher, Aryan Benevolent Home (ABH) Chatsworth and ABH Clayton Gardens.

### Study population

This was part of a larger study to obtain data on the effect of a group exercise programme on the physical, psychological, social functioning and immune health status of older persons aged 60 years and above living in low-income aged care facilities. The ageing population in South Africa is increasing, and it has been shown that South African adults have a high prevalence of inactivity, which in terms of attributable death ranked 9th, compared to other risk factors.^25^ Regular exercise is known to minimise the physiological effects of a sedentary lifestyle among older persons and increase active life expectancy by limiting the development and progression of chronic disease and disabling conditions.^25^ Homes that provide care for the aged from low-income groups in the eThekwini district are not known to have the resources to offer a structured, supervised exercise programme for their residents who stand to gain from this activity. Only basic medical and nursing are on offer. This article derives from the baseline ophthalmic data gathered on the functional health status of the participating residents from low-income homes.

These five low-income aged care facilities were randomly selected from a listing of 19 facilities that cater to the aged from low-income groups located within a 20 km – 30 km radius of the Durban Central Business District. The list was obtained from the Department of Social Development. Randomisation was done by putting the names of 19 low-income aged care facilities in a hat and randomly selecting five low-income aged care facilities. The five facilities selected represented at least 30% of old age residents in low-income facilities. These facilities cater for people who cannot afford to pay for private residential care acilities, often coming from lower income families for whom government pensions are the main source of income. Low-income homes for the aged are operated by NGOs. These homes are subsidised by the government through the Department of Social Development and, additionally, rely heavily upon donor funding to ensure sustainability. Basic nursing and medical care are provided at these institutions. These include blood pressure and glucose-level monitoring, administration of medication and a few facilities offer wellness programmes. These services are supplemented by healthcare offered at public hospitals. The average age of residents is between 65 and 75 years and the male:female ratio is 5:6.

Volunteers meeting the study’s inclusion/exclusion criteria were invited to a pre-selection screening process to determine eligibility, after which all eligible participants were invited on to the programme. Thus, no formal sample size was calculated at the start of the study. Inclusion criteria were the following: participants aged 60 years or older, independent in their activities of daily living. The exclusion criteria were the following: individuals under the age of 60, those with any disease or condition that excluded participation in the examination and those who were unable to give consent.

### Data collection

Socio-demographic data were collected for age, race, gender, marital status and year of last eye examination. To establish the prevalence and causes of VI and blindness, each participant underwent distance and near VA measurements, anterior and posterior segments examination, auto-refraction, and direct and indirect ophthalmoscopy. Intraocular pressure (IOP) was measured with a hand-held Perkins applanation tonometer. Visual acuity was measured in both eyes by a trained research optometrist with ocular diagnostic qualifications using a retro-illuminated logMAR chart with Tumbling-E optotypes (Precision Vision, La Salle, IL, USA) at a distance of 4 m. Anterior segment evaluation was performed with a penlight torch and a hand-held portable slit lamp. Posterior segment evaluation was done with a direct ophthalmoscope as well as a head-mounted binocular indirect ophthalmoscope. The presenting VA was recorded with the participant wearing his or her habitual optical correction. If no letter could be identified at 4 m, the participant was moved to distances of 3 m, 2 m or 1 m, consecutively. If no letter could be identified at all, VA was examined as counting fingers, hand movements, perception or no perception of light. Visual impairment was based on presenting VA and the WHO classification.^26^ Table 1 shows the categories and classification of VI used in the study.

**TABLE 1:** Visual acuity ranges and classification of visual impairment according to the World Health Organization classification.^26^

Snellen VA	VA (logMAR)	Classification
≥ 6/18	≥ 0.5	Normal/no visual impairment
< 6/18−6/60	< 0.5−1.0	Moderate visual impairment
< 6/60−3/60 (6/120)	< 1.0−1.30	Severe visual impairment
< 3/60	< 1.30	Blind

Note: Moderate and severe visual impairment constitute low vision.

VA, visual acuity; logMAR, logarithm of the minimum angle of resolution.

Cataract was regarded as the main cause of VI in an eye with significant cataract, which obscured the posterior segment. Uncorrected refractive error was defined as the presence of presenting VA < 6/18, which improved to 6/18 or better with the use of a pinhole. Posterior segment diseases were considered as the cause of VI in cases where there was no evidence of media opacity and VA did not improve with a pinhole. Participants with pinhole-corrected VA <6/18 in either eye underwent further examination using a direct ophthalmoscope to ascertain the cause of vision loss. If the cause of VI could not be determined by examination with direct ophthalmoscopy, the pupil was dilated and assessed with a binocular indirect ophthalmoscope. Amblyopia was considered as a cause of VI if corrected VA was < 6/18 with no obvious pathology or structural anomaly of the eye. As per the guidelines provided by Gilbert et al.,^27^ where there was more than one cause, the condition that could be most easily corrected or treatable was considered as the cause of VI.

### Data analysis

Data were analysed using the descriptive and inferential statistics of the Statistical Package for Social Sciences (SPSS) version 21 (IBM Corp., Armonk, NY 2012). Descriptive statistics (range, mean and standard deviation) were used to describe the cohort and the visual values. The relationship between VI and age was tested for significance using the Chi-squared test, with a *p*-value of < 0.05 being considered significant at a 95% confidence interval.

### Ethical consideration

Ethical approval to conduct the study was obtained from the University of KwaZulu-Natal (UKZN) Biomedical Research Ethics Committee (BE 080/14). The study adhered to the tenets of the Declaration of Helsinki involving human subjects. Written informed consent was obtained from each participant. Permission to conduct the study was also obtained from the Department of Social Development, KwaZulu-Natal province, and the management of old age facilities. Each participant was informed of the purpose of the study. All participants were informed that their willingness to participate in this research study was totally voluntary and that they were free to withdraw from the study at any point should they wish to do so. Confidentiality and anonymity were ensured as participants were identified only by reference numbers. During and after the study project, all the data and related documentation were kept securely in the office of the researcher in a locked cupboard. The data will be destroyed by shredding 5 years after the completion of the study. The electronic version of the data will be stored in a password-protected computer and deleted after 5 years of the completion of the study.

## Results

### Socio-demographic characteristics

A total of 118 older adults took part in the study. Their ages ranged from 60 to 88 years, with a mean age of 73.3 ± 4.6 years. There was no statistically significant difference between the mean age of the male (74.6 ± 3.8 years) and female participants (73.1 ± 4.9 years) (*t*-test, *p* = 0.33) (Table 2).

**TABLE 2:** Socio-demographic profile of the participants.

Characteristic	Subcategory	Number	Percentage
Location/area	TAFTA on Ridge	20	16.9
	TAFTA Ray Hulette	23	19.5
	TAFTA Mary Asher	25	21.2
	ABH Chatsworth	32	27.1
	ABH Clayton Gardens	18	15.3
Age (years)	60−69	42	35.6
	70−79	48	40.7
	80−89	28	23.7
Gender	Female	95	80.5
	Male	23	19.5
Race	Indian	77	65.3
	White	24	20.3
	Mixed race	15	12.7
	African	2	1.7
Marital status	Widowed	65	55.1
	Never married	20	16.9
	Married	16	13.6
	Divorced	15	12.7
	Not reported	2	1.7
When was your last eye examination	Within past year	19	16.1
	Between 1 and 2 years ago	24	20.3
	More than 2 years ago	45	38.1
	Never had an eye examination before	30	25.4

TAFTA, The Association for the Aged; ABH, Aryan Benevolent Home.

### Refractive error

Myopia 30.1% (*n* = 25) was the most common type of refractive error, followed by hyperopia 23.3% (*n* = 19) and myopic astigmatism 16.7% (*n* = 14). The mean spherical equivalent refractive error was −0.41 ± 1.7 D (range, –5.25 D to +14.50 D) in the right eye and −0.65 D ± 2.08 D (range, –3.50 D to +5.00 D) in the left eye. There were no cases of amblyopia in this study.

### Prevalence of visual impairment and blindness

Based on presenting distance VA, 46.6% (*n* = 55) (95% confidence interval [CI] = 0.0−53.2) had moderate VI (< 6/18−6/60), 10.2% (*n* = 12) (95% CI = 0.0−35.1) had severe VI (< 6/60−3/60) and 6.8% (*n* = 8) (95% CI = 0.0−32.8) were blind (VA < 3/60). After refractive correction, 33.1% (*n* = 39) (95% CI = 0.0−50.9) had moderate VI, 5.9% (*n* = 7) (95% CI = 0.0−32.2) had severe VI and 5.1% (*n* = 6) (95% CI = 0.0−31.6) were blind (Table 3).

**TABLE 3:** Presenting visual acuity in the better eye and visual acuity after best-corrected refraction.

VA (Snellen)	VA (logMAR)	*n*	%	95% CI
**Presenting VA in the better eye**				
≥ 6/18	≥ 0.5	43	36.4	0.0−53.2
< 6/18−6/60	< 0.5−1.0	55	46.6	0.6−59.9
< 6/60−3/60	< 1.0−1.30	12	10.2	0.0−35.1
< 3/60	< 1.30	8	6.8	0.0−32.8
**VA after refraction**				
≥ 6/18	≥ 0.5	66	55.9	6.2−66.1
< 6/18−6/60	< 0.5−1.0	39	33.1	0.0−50.9
< 6/60−3/60	< 1.0−1.30	7	5.9	0.0−32.2
< 3/60	< 1.30	6	5.1	0.0−31.6
**Total**	**-**	**118**	**100.0**	**33.8**−**93.8**

VA, visual acuity; CI, confidence interval.

95% CI are calculated using the Wilson approximation; negative values are truncated to 0.0.

Using the presenting VA in the better eye, the prevalence of VI and blindness was 63.6% (*n* = 75), which reduced to 44.1% (*n* = 52) after refractive correction, a reduction of 19.5% (*n* = 23), which was statistically significant (*p* < 0.05). Following refractive correction, the prevalence of low vision (VA < 6/18 to ≥ 3/60 in the better eye) was 39% (*n* = 46), of which 33.1% (*n* = 39) had moderate VI (< 6/18–6/60) and 5.9% (*n* = 7) had severe VI (< 6/60–6/120). The prevalence of blindness was 5.1% (*n* = 6) (Table 3).

### Causes of non-refractive visual acuity and blindness

The number, percentages and causes of non-refractive VI and blindness are shown in Table 4. The prevalence of VI and blindness in females was significantly higher than in males (*p* < 0.05). The prevalence of VI and blindness was significantly associated with age (*χ*^2^ = 16.02, *df* = 08, *p* < 0.05). The increasing prevalence of VI and blindness with increasing age was statistically significant in both men and women (both *p*-values < 0.05).

**TABLE 4:** Causes of non-refractive visual acuity and blindness.

Cause	Number	Percentage
Cataract	30	54.5
Corneal opacity	11	20.0
Retinopathy (diabetic and hypertensive retinopathies)	6	11.0
Glaucoma	4	7.3
Age-related macular degeneration	2	3.6
Retinal degeneration and dystrophy	2	3.6
**Total**	**55**	**100.0**

## Discussion

Although several studies^5,6,7,11,23^ have shown that VI is common in the older population, and that this risk increases rapidly with advancing age, most surveys of ocular diseases do not include low-income aged care facilities in South Africa, which was the setting of this study. Data on the prevalence and causes of VI are important for health authorities to plan and implement eye care services in this group. The findings of this study showed that much of the VI is preventable and is because of correctable or treatable conditions including refractive error and cataract. Provision of refractive or optical correction and cataract surgery can significantly reduce the prevalence of VI and blindness in old age facilities. The study showed that the prevalence of VI and blindness in older people residing in low-income old age homes of Durban, South Africa, is high. This is similar to the high prevalence of VI and blindness reported in old age facilities in Nepal^23^ (59.1%) and the United States^11^ (67%).

In the current study, there were more females than males in the old age homes. This gender distribution is similar to other reports by Eichenbaum et al.^6^ in New York City; Lamoureux et al.^8^ in Victoria, Australia; Elliott et al.^9^ in Alabama, the United States; and Dev et al.^23^ in Kathmandu Valley, Nepal. This could be because of the husbands passing away at an earlier age.

The study indicates that many elderly people had their last eye examination 2 or more years ago, with one-quarter reporting that they had never had their eyes tested. It has been well-documented that ‘ageing is associated with an increased rate of VI and eye diseases, some of which are potentially blinding’.^24^ Therefore, the government should provide regular eye examinations in these facilities to prevent unnecessary VI and blindness in older persons. This is particularly important as many visual problems that affect the elderly (particularly those of gradual onset such as specific types of glaucoma) are symptomless, many of which can be treated or corrected if identified early.^24^

The higher prevalence of myopia (mean = −0.41 ± 1.7 D, range, –5.25 D to +14.50 D) in the right eye and (mean = −0.65 D ± 2.08 D, range, –3.50 D to +5.00 D) in the left eye may have been because of the increased prevalence of cataract^23^ among the study participants. Only one type of cataract is associated with myopia in its early stages, and therefore the high prevalence of myopia may be more a reflection of the racial preponderance of the participants. For example, Dev et al.^23^ reported a mean spherical equivalent refractive error of −0.35 ± 2.86 D (range, −18.00 D to +12.00 D) in the right eye and −0.58 ± 2.92 D (range, − 22.00 D to +12.00 D) in the left eye among the older adults living in residential care centres of Kathmandu Valley, Nepal. The Nepalese may be racially very close to the current sample population, hence the high degree of myopia. The prevalence of VI and blindness significantly reduced following optical correction. Although refractive errors can be easily corrected by an eye examination and the provision of a pair of spectacles, many people in South Africa remain visually impaired as they do not receive eye care services.^24,28^ Researchers^24,28^ have suggested that this may be because of a shortage of eye care personnel, poor accessibility of the services or an inability to afford the service cost. Studies by Tielsch et al.^7^ in Baltimore, USA; Waked et al.^5^ in Lebanon; and Dev et al.^23^ in Nepal have also reported that the prevalence of VI reduced after best optical correction in low-income aged care facilities. This suggests that uncorrected refractive error is one of the most common causes of VI and blindness in old age persons in most developed and developing countries.

The high prevalence of common age-related ocular conditions is a reflection of the inadequacy of eye care services in the low-income aged care facilities studied. If adequate access to diagnostic and treatment services for retinopathies and glaucoma are in place, the VI from these conditions can be reduced. Although macular diseases to some extent may not be treated significantly to completely restore vision even with access to high-level care, low vision rehabilitation may be effective in improving functioning for people with these diseases. This finding suggests the need for the provision of vision screening, refraction and eye examinations, low vision rehabilitation and referrals to eye hospitals for those needing further assessment and management. Although old age home-based studies on VI in South Africa could not be found in the literature, the findings in this study reflect those of previous population studies^4,29^ among the elderly in the country, which found that cataract and posterior segment diseases were the leading causes of non-refractive VI and blindness. For example, a study conducted by Cockburn et al.^4^ reported that posterior segment diseases accounted for about 57% of VI among non-residential care adults aged 50 years and older in Cape Town, South Africa.

Increasing VI with age is not only peculiar to those in aged care facilities but all elderly populations. Being female was also associated with an increased risk of vision loss (*p* < 0.05). Cockburn et al.^4^ suggested that gender discordance in the prevalence of VI could be because of longer life expectancy among women, as many eye diseases are age related, and they appear to have an increased susceptibility to certain ophthalmic conditions. However, our results do not support this, as there was no difference in age between the males and females. Pascolini and Mariotti^1^ reported that in every region of the world, females have a significantly higher risk of developing VI than their male counterparts. This was similar to the report by Tielsch et al.,^7^ where females had higher rates of VI in their study of older adult residents in Baltimore. However, Eichenbaum et al.^6^ in New York City and Dev et al.^23^ in Nepal reported no significant gender difference in the prevalence of VI.

### Strengths and limitations of the study

This was a novel study, and it provides a comprehensive basis for establishing future studies in other regions. However, the findings in this study could not be directly compared with results of any surveys of elderly adults in old age homes elsewhere in Africa because of the absence of such studies, as indicated by rigorous literature search. One of the major weaknesses is that it is based on a small population in Durban and therefore not generalisable even to the elderly population in low-income aged care facilities, especially because of the racial composition of the study populations, which is significantly different from that of the general South African elderly population. In addition, this study is not generalisable to the Durban population of old age home residents as only the low-income homes were included in the study.

### Recommendations

#### Policy-makers

- The high prevalence of treatable conditions suggests the need to advocate for policy formulations on regular eye testing and referral of residents in these facilities.

#### Healthcare providers

- Healthcare providers such as optometry students can provide routine eye care screening for prompt identification of ocular conditions in old age home residents as part of their community outreach programme.

#### Aged care service providers

- Aged care service providers should train their staff at old age homes to identify and suspect visual conditions and their impact on daily functioning.

#### Residents of low-income aged care homes and their families

- Residents of low-income aged care homes and their families should be educated about the need and importance of regular eye testing and the implications of delayed eye care requirements.

#### Future studies

- The study needs to be replicated on a larger, more representative sample.

## Conclusion

The prevalence of VI among the older adults living in low-income old age homes in Durban is high, with refractive error and cataract being the leading cause of VI. Visual impairment can be significantly reduced by availing optical and rehabilitative low vision services, and providing cataract surgery to residents of low income old age facilities.
